# Identification and characterization of a stachyose synthase gene controlling reduced stachyose content in soybean

**DOI:** 10.1007/s00122-015-2575-0

**Published:** 2015-07-16

**Authors:** Dan Qiu, Tri Vuong, Babu Valliyodan, Haiying Shi, Binhui Guo, J. Grover Shannon, Henry T. Nguyen

**Affiliations:** Division of Plant Sciences, National Center for Soybean Biotechnology (NCSB), University of Missouri, Columbia, MO 65211 USA; Division of Plant Sciences and NCSB, University of Missouri, Portageville, MO 63873 USA

## Abstract

**Key message:**

**We identified and characterized a mutant of soybean stachyose synthase gene controlling reduced stachyose content which benefit the soybean seed composition breeding program in the future.**

**Abstract:**

It has been shown that in soybean, increased sucrose and reduced raffinose family oligosaccharides would have a positive impact on the world’s feed industry by improving digestibility and feed efficiency. We searched for new sources of modified oligosaccharide content in a subset of the USDA Soybean Germplasm Collection and then identified plant introduction (PI) 603176A as having ultra-low stachyose content (0.5 %). We identified a 33-bp deletion mutant in the putative stachyose synthase gene (*STS* gene, *Glyma19g40550*) of PI 603176A. A co-dominate indel marker was successfully developed from this 33-bp deletion area and was genetically mapped into two *F*_2:3_ populations and a *F*_4:5_ population, which associated with low stachyose content in the progeny lines. These observations provided strong evidence that the *STS* gene is responsible for stachyose biosynthesis in the soybean plant. Expression of the *sts* gene remained at the normal level, suggesting the loss of function in the gene is due to defective protein function. This gene-based perfect genetic marker for low stachyose content can be useful for marker-assisted selection in soybean molecular breeding programs.

**Electronic supplementary material:**

The online version of this article (doi:10.1007/s00122-015-2575-0) contains supplementary material, which is available to authorized users.

## Introduction

Soybean seed is a major food source providing protein, oil, carbohydrates, secondary metabolites, and other nutrients to humans and animals. The seed is comprised of on average 40 % protein, 20 % oil, and 33 % carbohydrates, of which up to 16.6 % of the total carbohydrates are soluble sugars (Hymowitz and Collins 1974). The major components of the soluble sugars are glucose, fructose, sucrose, raffinose, and stachyose. The amount of major soluble sugar components among soybean germplasm varies; e.g. sucrose 1.5–10.2 %, stachyose 1.4–6.7 %, and raffinose 0.1–2.1 % of the total dry matter (Hou et al. 2009; Hymowitz and Collins 1974).

Raffinose is a trisaccharide that can be found in the cotyledons, seed coats, and hypocotyls (Bentsink et al. 2000). Stachyose is a tetrasaccharide, which is recognized as an important transport carbohydrate in a large number of woody plants, cucurbits and legumes (Peterbauer et al. 1999). The most common raffinose family oligosaccharides (RFOs) are trisaccharide raffinose, tetrasaccharide stachyose and the pentasaccharide verbascose (Minorsky 2003). RFOs can act as reserve carbohydrates, membrane stabilizers and stress tolerance mediators (Bentsink et al. 2000; Elsayed et al. 2013; Karner et al. 2004; Van den Ende 2013). Since soybean meal is a common source of protein for livestock (Meis et al. 2003) and humans (Guimaraes et al. 2001), soybean digestibility is important. Higher concentration of RFOs in seeds is one of the major problems in the efficient utilization of soybean for human food and animal feed applications. In soy meal fed monogastric animals poor digestibility of RFOs causes a reduction in metabolizable energy and an increase in flatulence and diarrhea. In addition to their indigestibility, raffinose and stachyose can cause diarrhea that may increase digesta passage rate and decreased digestion and absorption of dietary nutrients (Parsons et al. 2000).

Specific galactosyltransferases enzymes (e.g. raffinose synthase, stachyose synthase, etc.) catalyze the reaction towards biosynthesis of raffinose and stachyose from sucrose. A number of gene sequences have been annotated as raffinose synthases, but biochemical confirmation and molecular characterization have only been completed for maize (Zhou et al. 2012), pea (Peterbauer et al. 1999), soybean (Dierking and Bilyeu 2008; Skoneczka et al. 2009) and rice (Li et al. 2007). In *Arabidopsis thaliana*, a knock-out mutation of raffinose synthase (*RS*) or the overexpression of galactinol synthase (GolS) caused the reduction of leaf raffinose levels when compared to wild type (Zuther et al. 2004). To our knowledge, there was no evidence in the literature that stachyose synthase exists in soybean. The only published experiments indicate that an adzuki bean stachyose synthase is capable of catalyzing the synthesis of both stachyose and verbascose (Peterbauer et al. 1999, 2002). Overexpression of *STS* from adzuki bean (*Vigna angularis*) in *Arabidopsis* had accumulated stachyose upon cold acclimation (Iftime et al. 2011).

The reduction in oligosaccharide levels in soybean meal could increase the amount of soy proteins in rations (Hartwig et al. 1997). In past years, many efforts have been made to evaluate existing soybean germplasm and mutagenized materials aiming for the improvement of digestible carbohydrates and better nutritional factors. Sebastian et al. (2000) screened bulk seed from approximately 8000 individual M3 generation plants and the USDA Soybean Germplasm Collection, and identified two types of modified carbohydrate profile soybean seeds. Soybean accession PI 200508 was identified as having reduced levels of RFOs and elevated levels of sucrose (Kerr and Sebastian 2000). Initial characterization of this PI was carried out by Hitz et al. (2002). Later, it was reported that PI 200508 allele of *RS2* (raffinose synthase) was associated with the increased sucrose and low raffinose and stachyose seed phenotype (Dierking and Bilyeu 2008; Skoneczka et al. 2009). The intellectual property of the above soybean line limits the development of these traits in public breeding programs.

In an effort to discover new sources of modified sucrose and RFO’s, a subset of over 650 soybean germplasm accessions with maturity group (MG) ranging from III to V were evaluated at the University of Missouri. This initial screening has identified several potential PIs with lower raffinose and stachyose content and higher sucrose level. These PIs were evaluated for sugar composition stability in different growing environments. Here we report the identification of a stachyose synthase gene controlling reduced stachyose content in soybean PI 603176A (0.5 %), from which a co-dominant genetic marker was successfully developed and genetically mapped. This gene-based genetic marker can be useful in molecular soybean breeding programs, aiming towards breeding of modification of sucrose and RFOs in soybean.

## Materials and methods

### Screening of seed raffinose and stachyose content in 650 soybean germplasm

A total of 650 soybean accessions (from MG III to V) were requested from GRIN (Germplasm Resources Information Network) (http://www.ars-grin.gov). The seed raffinose and stachyose content of these 650 accessions were quantified by standard HPLC method which was described at the below “Sugar content quantification of soybean seeds by HPLC”.

### Population development

Two newly identified soybean accessions with low RFOs (stachyose + raffinose), PI 603176A and PI 594012, along with elite lines with regular RFO’s content, S07-5049 and S05-11482, were utilized to develop genetic populations in this study. A cross of line S07-5049 and PI 603176A was made in the summer of 2010 at the Bradford Research and Extension Center (BREC), University of Missouri, Columbia, MO. *F*_1_ hybrid of this cross were planted in Costa Rica and *F*_2_ seeds were planted at BREC in summer of 2011. One hundred and thirty-one resulting *F*_2:3_ progenies derived from this cross were screened for seed oligosaccharide content using HPLC system (Hou et al. 2009). A second population of 70 *F*_2:3_ progenies was developed from a PI 594012 × PI 603176A cross. In addition, a third population of 82 *F*_4:5_ advanced inbred lines developed from a S05-11482 × PI 603176A cross was subsequently employed for confirmation tests. Both these two populations were planted in Costa Rica and seeds were shipped back for further analysis.

### DNA extraction and sequencing of stachyose synthases gene (*STS* gene)

Genomic DNA was isolated from young leaf tissue of the parents and progeny plants using a standard CTAB protocol (Vuong et al. 2010). The DNA concentration was quantified with a spectrophotometer (NanoDrop Technologies Inc., Centreville, DE) and diluted to a concentration of 50 ng/µl for polymerase chain reaction (PCR) amplification to amplify the target regions or for sequencing.

Soybean sequences were obtained after PCR and either direct sequencing or cloning followed by sequencing (Wu et al. 2010). PI 603176A, S07-5049, and their progeny lines carrying the *STS* alleles were sequenced from PCR products, which were amplified with primers that were intronic, flanking exonic sequences.

### Allele-specific molecular marker assay development

PCR amplifications were performed in 25 µl final volume on the Eppendorf 96-well thermal cyclers with three primers: Primer_GCF_G:GCGGGCAGGGCGGCAGGGTGATGGGAGATTCCTTG, Primer_GCF_T:GCGGGCAGGGTGATGGGAGATTCCTTT, Primer_common: ACTCAAAAGCAACATCAGAACCAT (Eppendorf AG, Germany). Each reaction contained 40–50 ng of genomic DNA, 0.13 µM of forward primer and reverse primer, 0.2 mM of each dNTP, and SYBRGreen mix solution (GenScript Corp., Piscataway, NJ). The thermal cycler program was performed at 95 °C for 5 min followed by 35 cycles of 95 °C for 20 s, 60 °C for 20 s, and 72 °C for 20 s. Melting curve from 60 to 85 °C, with readings taken every 0.1 °C.

### Sugar content quantification of soybean seeds by HPLC

Seed sucrose and RFO contents of soybean lines in each genetic population were quantified. Approximately 1 g of dried seed from each line was ground to fine powder. 0.1 g ground soybean powder was air-dried for 2 days, followed by the addition of 0.9 ml of HPLC grade water in a 2-ml centrifuge vial. Sample tubes were incubated at 55 °C for 20 min with 200 rpm agitation. Subsequently, 0.9 ml of 95 % acetonitrile was added and vortexed for 30 s, followed by centrifuging for 10 min, and filtered with a syringe and 0.45 µm filter. A sample solution of 100 µL was mixed with 400 µL of 65 % acetonitrile in an HPLC vial. Standard sugar melibiose (Sigma Chemical Co., St. Louis, MO) was used as the internal standard. A soybean cultivar, Williams 82, and a low-stachyose line, PI 200508, were included as checks in each extraction and quantification in order to monitor the consistency and accuracy of the tests.

### Expression analysis by quantitative RT-PCR

Primer sequences for the candidate genes are *STS*: 5′-GGGTGATGGGAGATTCC-3′ and 5′-CTCAAAAGCAACATCAGAACC-3.

The primers for the housekeeping gene, elongation factor 1α are 5′-CTGTAACAAGATGGATGCCACTAC-3′ and 5′-CAGTCAAGGTTAGTGGACCT-3′ (Czechowski et al. 2005). The real time polymerase chain reaction (RT-PCR) was performed using the QuantiTect SYBR Green RT-PCR Kit (Qiagen, Valencia, CA) in 10 μL reactions. The parameters for the one step RT and the PCR were as follows: reverse transcription at 50 °C for 30 min followed by 95 °C for 15 min, then 35 cycles of 95 °C for 15 s, 55 °C for 30 s, and 72 °C for 30 s with an ending hold at 4 °C. Experiments included control reactions lacking the reverse transcriptase enzyme to assess possible genomic DNA contamination.

## Results

### Screening of seed raffinose and stachyose content in the soybean germplasm

A total of 650 soybean accessions (from MG III to V) were screened for natural variation of seed sugar components. The frequency distribution of seed raffinose and stachyose content for 650 accessions are shown in Supplement S1. The initial screening identified a few lines with low raffinose and low stachyose content. Those lines were planted in different environments for the stability test. Subsequently, the lines with low raffinose and low stachyose content in more than two environments were selected for further genetic analysis (Table 1). PI 603176A showed a significant reduction in stachyose content from 5 to 0.5 % in different environments. Three genetic populations derived from this PI line were developed for genetic analysis and gene mapping.

**Table 1:** Seed RFO content and non-synonymous SNPs variation in RS2, RS3, RS4, and STS genes in the selected soybean lines

No.	Phenotypic data			SNPs variation										
	Sucrose (%)	Raffinose (%)	Stachyose (%)	RS2	RS2	RS2	RS2	RS3	RS3	RS3	RS4	RS4	STS	STS
SNPs				G512C	G1609A	G2000T	C2123A	C28G	G48T	C1105G	C197G	A1061G	T1895A	T2558G
AA				C171S	V537I	S667I	T708N	P10A	K16N	R369G	T66S	E354G	M632K	V853G
PI 603176A	4	3.31	0.55		Y			Y	Y	Y				
PI 594012	6.5	0.92	2.89		Y	Y	Y	Y	Y	Y			Y	
S07-5049	5.1	0.75	3.12											
S05-11482	4.5	1.31	4.52		Y			Y		Y				
PI 086006	2.4	0.7	3.6					Y	Y	Y		Y		
PI 424079	1.52	0.59	3.85		Y	Y	Y	Y	Y	Y			Y	
PI 437654	5.35	0.68	4.75		Y			Y	Y	Y	Y			Y
PI 200508	7.1	1.1	1.5	Y	Y			Y	Y	Y				
222-18-1^a^	7.5	0.13	0.58	Y	Y			Y	Y	Y		Y		
Williams 82	5.2	0.99	4.14											

Y means contain the SNP variation

^a^A soybean line with ultra-low stachyose and raffinose from the patent of Schillinger et al. 2011

Identification of the *STS* gene mutation in PI 603176A and single nucleotide polymorphism (SNP) variation in the soybean germplasm.

**Fig. 1:**
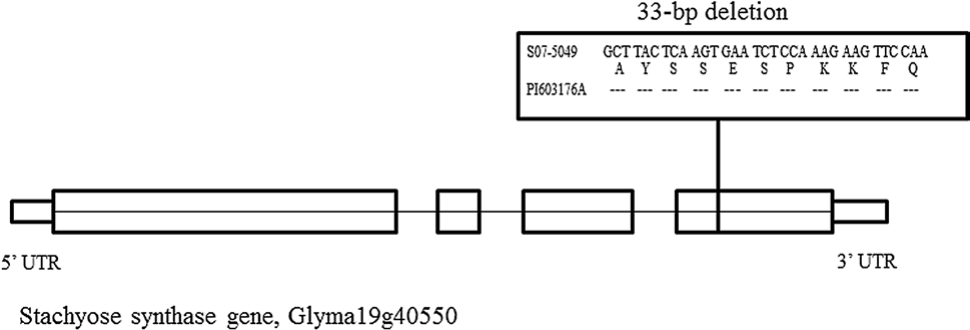
A 33-bp deletion in the 4th exon of PI 603176A stachyose synthase gene

The stachyose synthase gene (*STS*, *Glyma19g40550*) is a putative gene involved in a pathway which converts raffinose to stachyose. Besides that, the genes *RS2* (*Glyma06g18890*), *RS3* (*Glyma05g08950*), and *RS4* (*Glyma05g02510*) were confirmed to be involved with the raffinose metabolism. We have sequenced these four genes in PI 603176A along with other lines (Table 1). A number of SNP variations were identified in these four genes (Table 1). Interestingly, the sequence of the *STS* gene in PI 603176A showed that there is a 33-bp deletion in the exon 4 of this gene compared to an elite line S07-5049, which caused the 11 amino acid deletion in the protein (Fig. 1).

We also identified new non-synonymous SNPs, such as G2000T and C2123A in gene *RS2*, C197G in gene *RS4*, T1895A and T2558G in *STS* gene, which caused the amino acid changes (Table 1). The PI 594012 showed second lowest stachyose content at 2.89 % among the 650 germplasm accessions evaluated in this study. There were three non-synonymous SNPs in the *RS2* and *RS3* genes and one non-synonymous SNP in *STS* gene of PI 594012. In addition, two other PI lines, PI 424079 and PI 437654, also showed the lowest raffinose content with the various non-synonymous SNPs in these four genes. Further genetic analysis is needed for the functional confirmation of these SNP variation.

### Statistical analysis of seed stachyose content in a genetic population

**Fig. 2:**
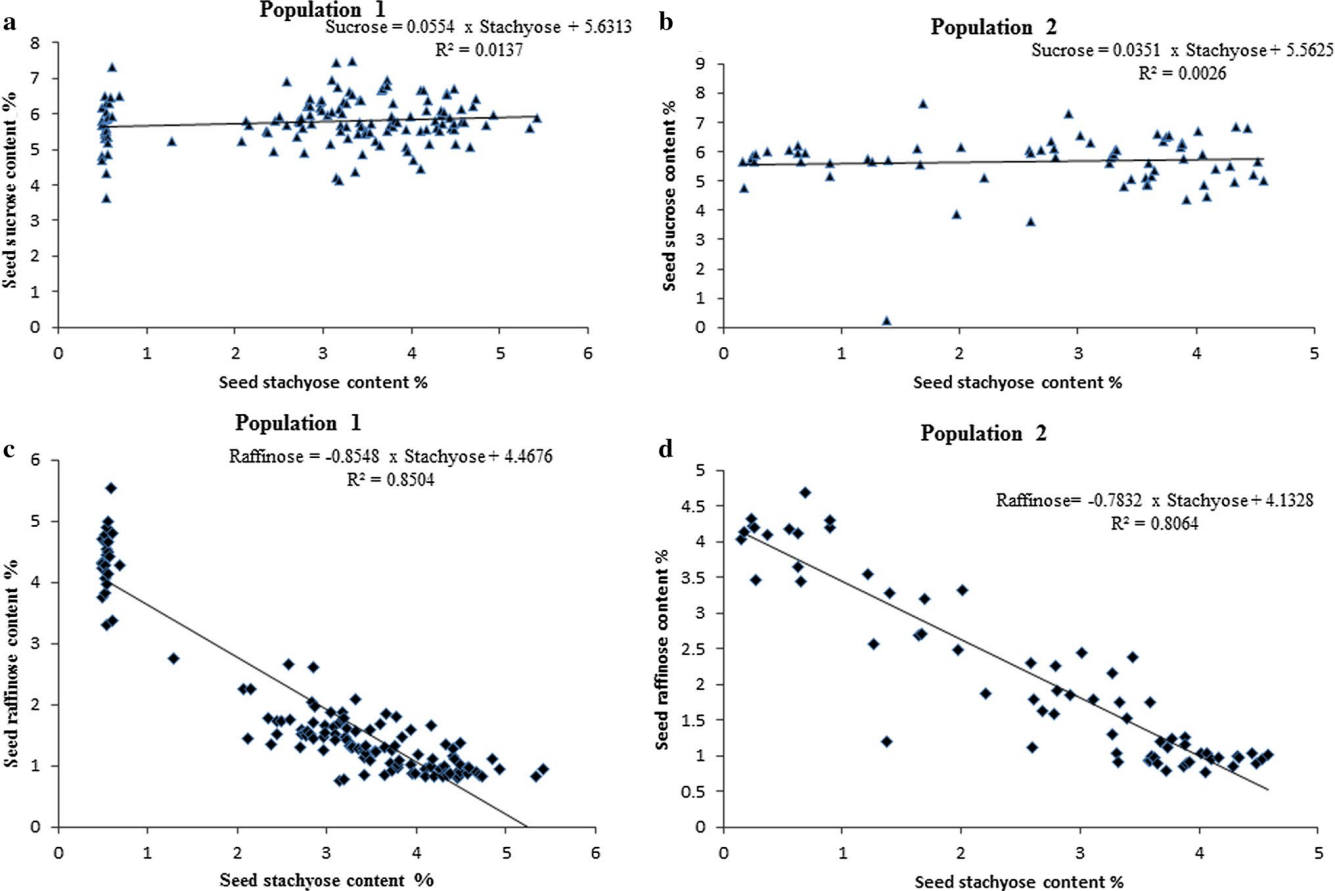
Scatterplot of percent seed sucrose and raffinose content (*Y*-axis) and percent seed stachyose content (*X*-axis) in Population 1 (S07-5049 × PI 603176A, **a**, **c**) and Population 2 (PI 594012 × PI 603176A, **b**, **d**). Sucrose values are represented by *triangles* and raffinose values by *diamonds*. Trend line equations are given at the *top right* of each plot

A new cross of PI 603176A and an elite soybean line S07-5049 with regular raffinose and stachyose content was made to produce an *F*_2:3_ population. PI 603176A has high raffinose content (3.31 %) and low stachyose content (0.55 %), while S07-5049 has regular raffinose (0.9 %) and stachyose content (4.6 %) (Table 2; Fig. 2). We have analyzed an *F*_2:3_ population derived from a cross between these two parental lines. The stachyose and raffinose content showed characterization of a Mendelian factor with a segregation ratio of 1:2:1 (*P* > 0.1). A significant negative correlation (*r*^2^ = −0.92, *P* < 0.001) was observed between raffinose and stachyose content in this genetic population (Table 3). In contrast, no significant correlation was found between sucrose content and raffinose/stachyose content in this population.

**Table 2:** Seed stachyose content (%) means and Chi square values in three soybean populations segregating for low seed stachyose content

No.	Parent				Population						
		PI	Raffinose (%)	Stachyose (%)	Genotype	Observed	Raffinose (%)	Stachyose (%)	Expected	*χ* ^2^	*P*
131 *F*_2:3_ lines	Female	S07-5049	0.75	3.12	*STS/STS*	33	0.77–1.23	3.27–5.42	33	2.03	0.361
	Male	PI 603176A	3.31	0.55	*STS/sts*	72	1.64–3.15	1.32–3.01	65		
	Ck	Williams 82	0.99	4.14	*sts/sts*	25	3.36–5.53	0.49–1.11	33		
					Total	131	0.77–5.53	0.49–5.42	131		
70 *F*_2:3_ lines	Female	PI 594012	0.92	2.89	*STS/STS*	20	0.76–0.98	3.72–4.57	18	2.04	0.686
	Male	PI 603176A	3.42	0.52	*STS/sts*	35	1.02–2.68	1.23–3.67	34		
	Ck	Williams 82	1.03	4.26	*sts/sts*	15	3.27–4.67	0.36–1.22	18		
					Total	70	0.76–4.67	0.36–4.57	70		
82 *F*_5_ lines	Female	S05-11482	1.31	4.52	*STS/STS*	36	0.56–0.98	3.52–5.03	38	0.105	0.948
	Male	PI 603176A	3.38	0.49	*STS/sts*	4	1.22–2.78	1.29–3.23	4		
	Ck	Williams 82	1.11	4.36	*sts/sts*	42	3.47–5.37	0.46–1.02	38		
					Total	82	0.56–5.37	0.46–5.03	80

**Table 3:** The significant negative correlation between the stachyose content and raffinose content in three soybean populations

R	S07-5049 × PI 603176A		PI 594012 × PI 603176A		S05-11482 × PI 603176A	
	Raffinose	Stachyose	Raffinose	Stachyose	Raffinose	Stachyose
Sucrose	−0.013	0.073	0.194	0.051	0.121	0.134
Raffinose		−0.925**		−0.898**		−0.938**

** Significant at *P* = 0.001

### The identification of the *STS* gene

**Fig. 3:**
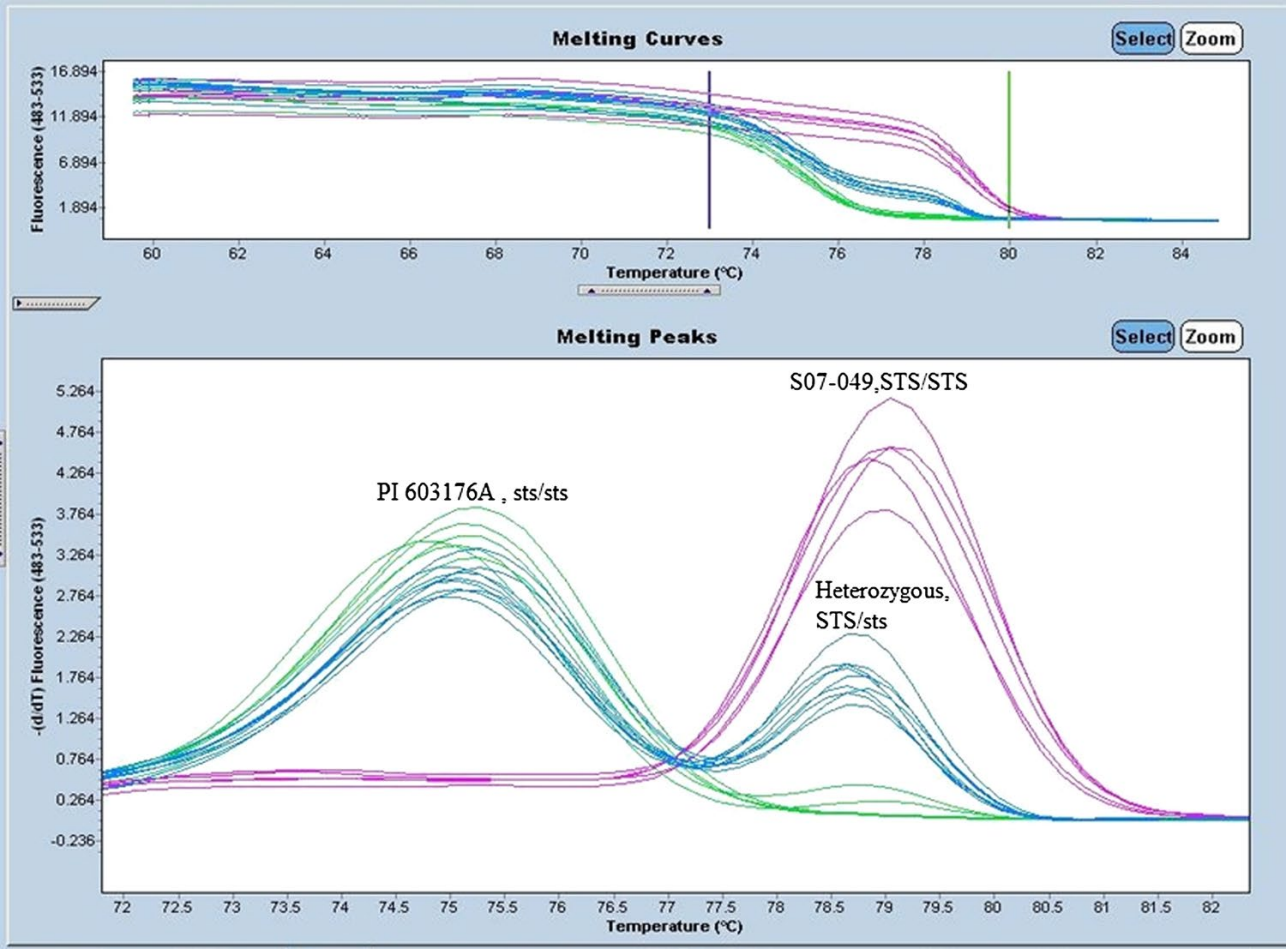
SimpleProbe marker assays were developed based on a 33-bp deletion associated with low stachyose content in the S07-5049 × PI 603176A population. The homozygous *STS* genotype gives the peaks at 79 °C and the homozygous *sts* genotype gives the peaks at 75.5 °C. Heterozygotes, *STS/sts*, show both peaks

Statistical analysis of phenotypic data in the S07-5049 × PI 603176A population showed that a single gene plays key role in a pathway of raffinose to stachyose. We developed this deletion (*sts* genotype) into the Allele-Specific molecular marker assay (Fig. 3). The marker assay data from *F*_2:3_ plants for both candidate genes and data sets revealed 1:2:1 ratio (*P* < 0.05) for PI 603176A alleles (*sts*/*sts*): heterozygote (*STS*/*sts*): S07-5049 alleles (*STS*/*STS*) (Table 1). The association analysis of phenotypic data of raffinose and stachyose content and genotypic data determined a strong association of the RFOs content and the *sts* genotype. A significant positive correlation (*r*^2^ = 0.89, *P* < 0.001) was observed between stachyose content and the *sts* genotype. In contrast, a significant negative correlation (*r*^2^ = −0.86, *P* < 0.001) was observed between raffinose content and the *sts* genotype. Results confirmed the biochemical function of *STS* gene in converting raffinose into stachyose. The functional *STS* gene in S07-5049 converts raffinose into stachyose and causes low raffinose and high stachyose content in regular soybean seeds. The *sts* mutant in PI 603176A resulted in the seed’s inability to convert raffinose to stachyose and the accumulation of raffinose content, which as same as in the progeny lines.

### Expression analysis of *STS* gene by quantitative RT-PCR

**Fig. 4:**
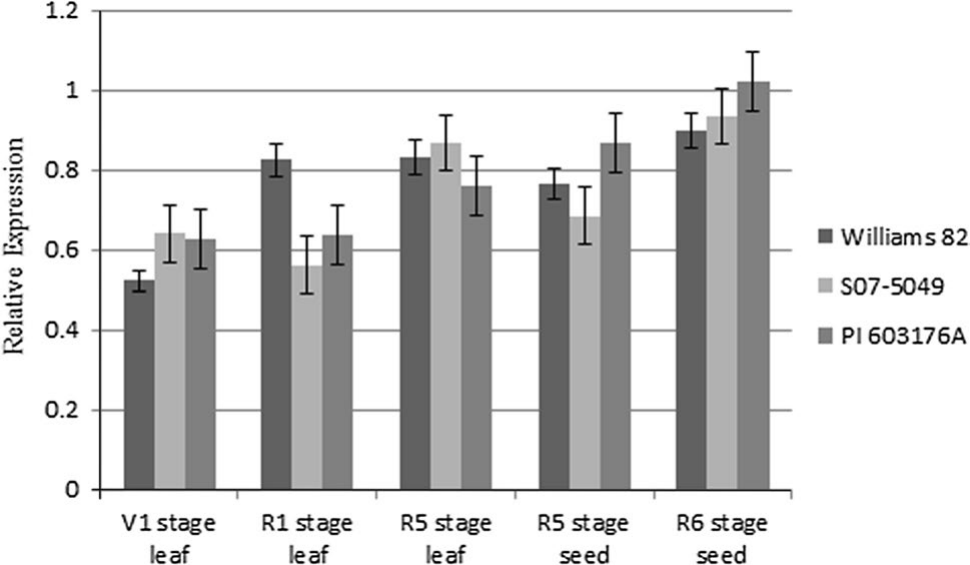
Relative expression of STS gene at different stages in cv. Williams 82, S07-5049, and PI 603176A determined by quantitative RT-PCR. V2 stage, two sets of unfolded trifoliate leaves. R1 stage, beginning flowering. R5 stage, beginning seed. R6 stage, full seed

Expression of the *STS* gene was determined using quantitative RT-PCR in cv. Williams 82, S07-5049, and PI 603176A. Leaf tissue from these three parental lines was collected at the vegetative stage V1, reproductive stages R1, and R5 growth stages and seed tissue was collected at the R5 and R6 growth stages (Fehr and Caviness 1977). The results indicated that the putative stachyose synthase genes have similar transcript levels in the three parental lines when compared to the housekeeping gene, elongation factor 1α (Czechowski et al. 2005). There is a slight increase of expression in sts genes of PI 603176A from the V1 to R6 growth stage (Fig. 4). Both *STS* and *sts* genes do not appear to be tissue specific or highly expressed in the developing seed tissues. Similar transcript levels as determined by quantitative RT-PCR, for *STS* and *sts*, and for all three lines, indicated that the difference in oligosaccharide content was unlikely to be a product of disruption in the transcriptional machinery, but rather a functional loss of protein.

### Analyzing oligosaccharide content in the two other populations and progeny lines

In order to test the stability of the *sts* mutant across the generation and environments, we selected three low stachyose *F*_2:3_ progenies for breeding purpose. Fourteen *F*_4_ lines were harvested in a greenhouse in May, 2013. The sequences and the genetic markers had confirmed the *sts* genotype in these 14 lines. Sugar analysis revealed that these 14 lines had low stachyose content (~0.5 %).

Moreover, over 70 *F*_2:3_ lines of the PI 594012 × PI 603176A population and 82 *F*_4:5_ lines of the S05-11482 × PI 603176A population were developed for the confirmation test. The phenotypic data and genotypic data were matched perfectly within progeny lines in these two populations (Table 2). The *sts* genotype is always associated with low stachyose content and high raffinose content.

### Phylogenetic relationships of different *RS* and *STS* proteins in plant genomes

**Fig. 5:**
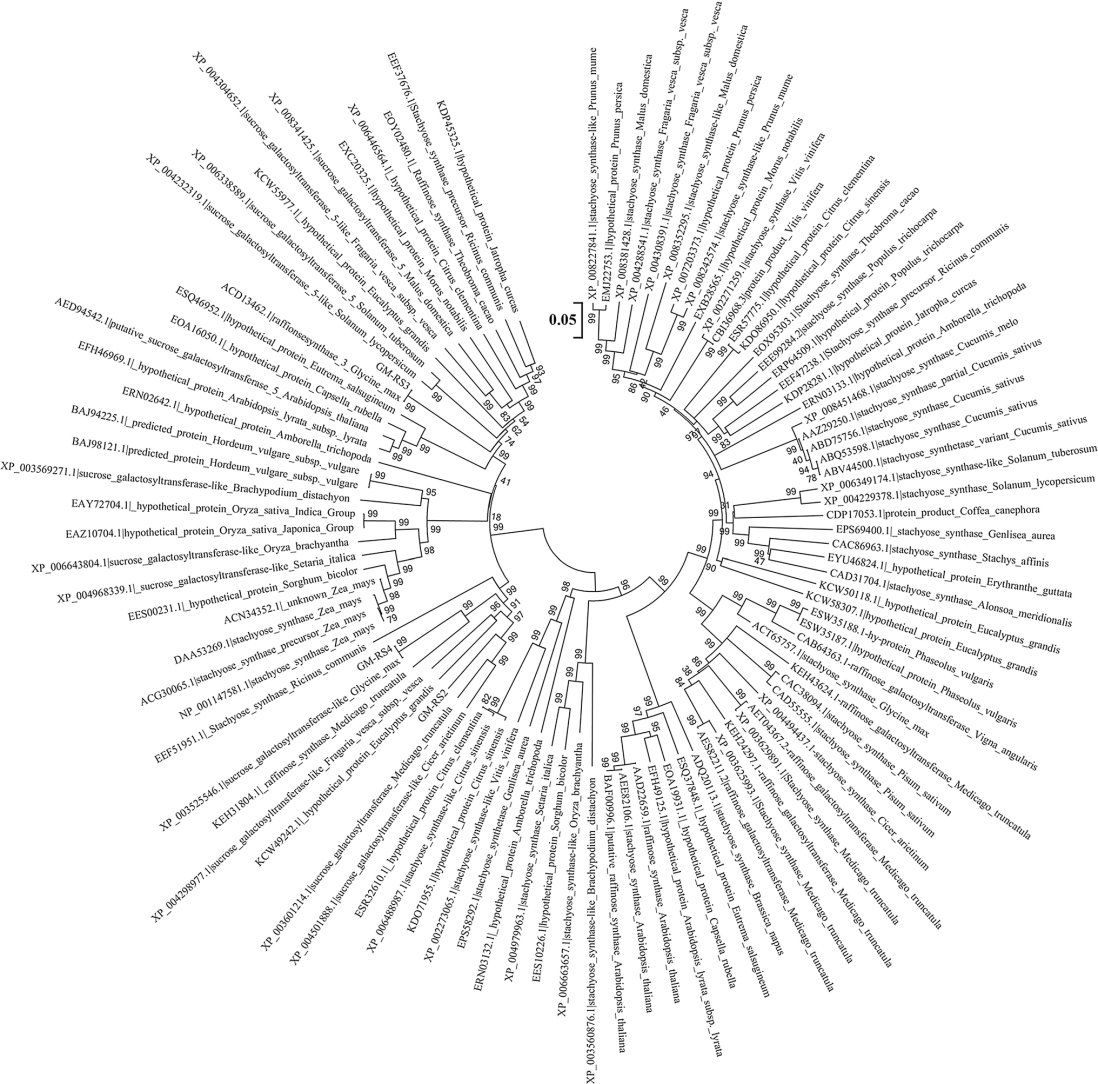
Phylogenetic relationships of 101 RS and STS proteins in 45 plant genomes using Bayesian analysis. Clades with 75 % posterior probability are displayed with *numbers above the lines*

Both *RS* and *STS* genes contain the same Pfam structure, PF05691 (Raffinose_synthase). Based on the EMBI (European Bioinformatics Institute) data base, 136 Eukaryote, 53 Bacteria and 21 Archaea species shared the PF05691 structure domain. About 101 *RS* and *STS* protein sequences from 43 plant genomes were selected to generate phylogenetic tree (Fig. 5). The soybean *STS* genes shared highest amino acid similarity with *STS* genes compared to species *Phaseolus vulgaris* and *Vigna angularis* at 87 %, following a similarity of 78 % in *Cicer arietinum* and *Medicago truncatula*. It is not surprising that the soybean *RS* and *STS* genes were grouped into different subclades due to only 45 % amino acid similarities between them. The sequence identity for soybean *RS2* and *RS3* is 65 %, for *RS2* and *RS4* is 70 %, and for *RS3* and *RS4* it is 62 % at the amino acid level, which is shown in the phylogenetic tree (Fig. 5).

## Discussion

We have successfully identified a 33-bp deletion mutant in the putative soybean stachyose synthase (*STS*) gene, characterized their expression in multiple tissues, developed and characterized the phenotype–genotype association in three segregating genetic populations. We confirmed the function of *STS* gene, which can convert raffinose into stachyose as part of raffinose metabolism (Dierking and Bilyeu 2008; Skoneczka et al. 2009). The *sts* mutant cut off the conversion of raffinose to stachyose, which caused a 90 % reduction of stachyose and the accumulation of raffinose content in soybean seeds. These data provided strong evidence that the *STS* gene was responsible for stachyose biosynthesis in the soybean plant. The *sts* genotype was always associated with low stachyose content (0.5 %), showing a significant reduction from regular stachyose content (5 %). This *sts* genotype mutant from PI 603176A associated with the low seed stachyose content was also confirmed in different progeny lines from three genetic populations which under different growing environments.

Raffinose and stachyose are tri-saccharides and tetra-saccharides, respectively. These two types of enzymes are involved mainly in the biosynthesis of RFOs. The raffinose synthase is the galactinol synthase, which generates galactinol from galactose and myo-inositol, the second one being galactosyltransferases (raffinose and stachyose synthases), which are responsible for incorporating the galactosyl group from galactinol into the oligosaccharide (Peterbauer et al. 1999, 2002). The *STS* sequences in adzuki bean, maize, and soybean share a high similarity with known *RS* sequences because both *RS* and *STS* contain the same Pfam structure (Raffinose_synthase, PF05691) (Peterbauer et al. 1999; Zhou et al. 2012). It is also notable that based on the EMBI database, 136 Eukaryote, 53 Bacteria, and 21 Archaea species shared the PF05691 structure domain, which might indicate the evolutionary history of the raffinose synthase gene. Nevertheless, the adzuki bean stachyose synthases are distinguishable from raffinose synthases by a characteristic central insertion of 70–80 amino acids (Peterbauer et al. 1999, 2002). Likewise, the amino acid sequence identity is 45 % for *STS* and *RS* genes in soybean and 42 % in pea (Dierking and Bilyeu 2008). The blast hit result from NCBI database shows that the *STS* genes in *Phaseolus vulgaris* and *Vigna angularis* have the highest amino acid similarity at 87 % when compared with soybean. This is followed by a 78 % similarity in *Cicer arietinum* and *Medicago truncatula*, which suggests that the *STS* genes in these species might have the same stachyose synthase function.

So far, the *RS2* mutant allele from PI 200508 was the main source for the selection of low raffinose and stachyose seed phenotype in soybean (Dierking and Bilyeu 2008). Typically, the seed raffinose content of the *RS2* allele remains at a regular level of 1 %, but the stachyose content drops to 1.5 %. It was reported that a soybean line, 222-18-1, exhibited ultra-low raffinose content (0.13 %) and stachyose content (0.58 %), and in which the *RS2* allele was combined with other *RS3* and *RS4* mutant alleles (Schillinger et al. 2011). This might suggest the *RS2*, *RS3*, and *RS4* alleles are three main enzymes responsible for converting galactinol into raffinose; however, the function of *RS3* and *RS4* are still unknown. The *sts* mutant allele in our study causes only at a level of 0.5 % stachyose content in seed. The *sts* genes are highly expressed in both leaf and developing seeds, which indicated that the 11-amino acids deletion might cause a functional loss of protein. There was still a production of 0.5 % stachyose content in the mutant. We speculate that this was possibly due to a partial function of the *sts* mutant or from the functional alleles of *RS3* and *RS4*.

Due to the increased use of soybeans and soy products in the feed industry, it is important to understand the nutritional and anti-nutritional components of soybean. Soy meal fed monogastric animals suffer from a reduction in metabolizable energy and an increase in flatulence and diarrhea (Coon et al. 1990). The *sts* allele was always associated with a low stachyose content (0.5 %), but at the same time it was associated with a high raffinose content (4.5 %) which preserves the total RFOs content at a regular level. Although the best combination level of raffinose/stachyose content for animal feeding has not been well determined, the *sts* allele can be combined with other *RS2/RS3/RS4* alleles to achieve a more nutritional combination of raffinose/stachyose content in soybean seeds. Molecular marker assays associated with the 90 % of stachyose content reduction were developed and validated in three mapping populations throughout different environments. This genetic marker will allow the incorporation of the desirable RFO traits into soybean varieties through molecular breeding.

### **Author contribution statement**

D.Q, B.V and T.D.V designed research; D.Q, B.V, T.D.V, B.G, H.S and J.G.S performed research; D.Q analyzed data and wrote the manuscript; T.D.V, B.V and H.T.N edited the manuscript; and H.T.N oversaw the project.

## Electronic supplementary material

Supplementary material 1 (tif 3572 kb)
