# Supplementary figures and images for: Identification and characterization of a stachyose synthase gene controlling reduced stachyose content in soybean
Source: Theor Appl Genet. 2015 Jul 16;128(11):2167–76. doi: 10.1007/s00122-015-2575-0 (PMC4624830; doi:10.1007/s00122-015-2575-0)

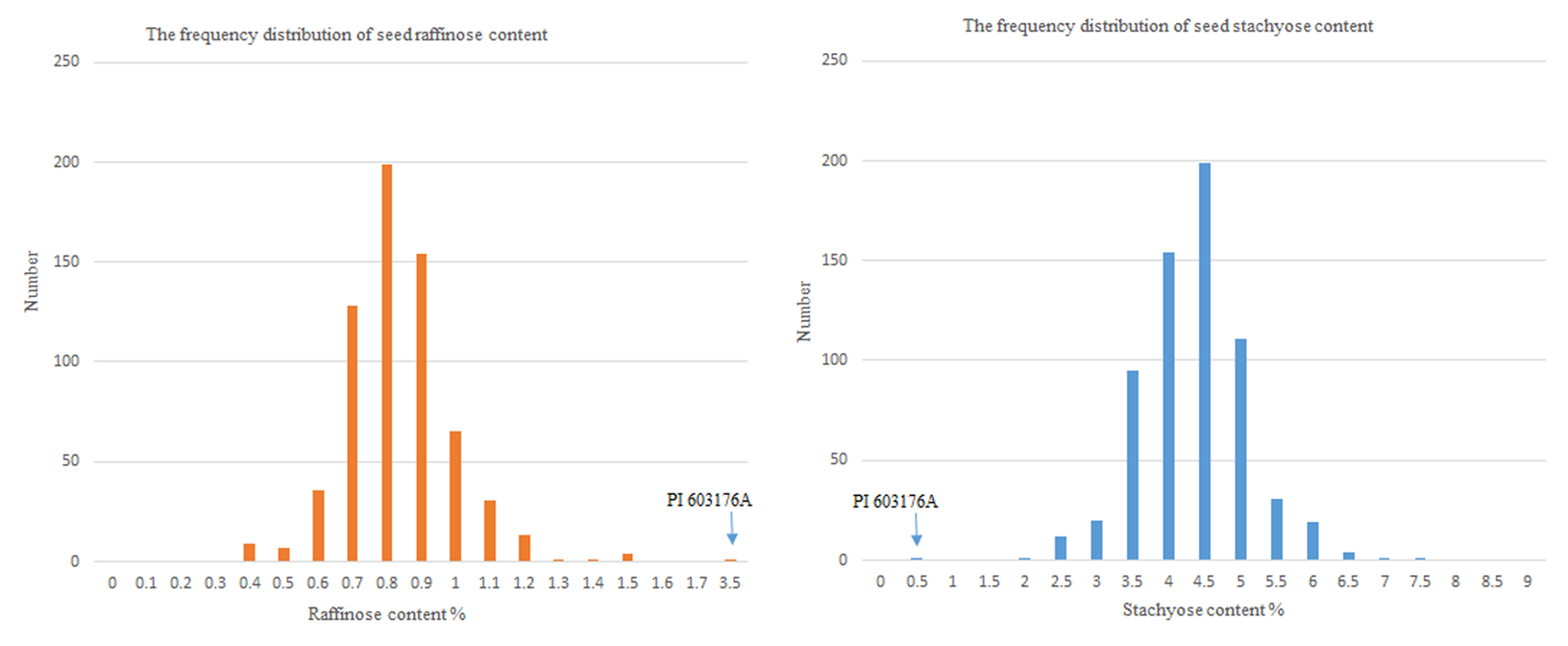

Supplement: Supplementary file 1 — Supplementary material 1 (tif 3572 kb) [file 122_2015_2575_MOESM1_ESM.tif]
